# Community voices: the importance of diverse networks in academic mentoring

**DOI:** 10.1038/s41467-022-28667-0

**Published:** 2022-03-25

**Authors:** Rocío Deanna, Bethann Garramon Merkle, Kwok Pan Chun, Deborah Navarro-Rosenblatt, Ivan Baxter, Nora Oleas, Alejandro Bortolus, Patricia Geesink, Luisa Diele-Viegas, Valeria Aschero, María José de Leone, Sonia Oliferuk, Rui Zuo, Andrea Cosacov, Mariana Grossi, Sandra Knapp, Alicia Lopez-Mendez, Elina Welchen, Pamela Ribone, Gabriela Auge

**Affiliations:** 1 Department of Ecology and Evolutionary Biology, University of Colorado, Boulder, CO USA; 2 ARG Plant Women Network, Buenos Aires, Argentina; 3 Instituto Multidisciplinario de Biología Vegetal (CONICET-UNC), Córdoba, Argentina; 4 Department of Zoology & Physiology, and Biodiversity Institute, University of Wyoming, Laramie, WY USA; 5 Hong Kong Baptist University, Hong Kong, China; 6 School of Environment and Sustainability, University of Saskatchewan, Saskatoon, Canada; 7 The University of West England, Bristol, UK; 8 Escuela de Salud Pública (Programa Doctorado en Salud Pública), Universidad de Chile, Santiago, Chile; 9 Donald Danforth Plant Science Center, St. Louis, MO USA; 10 Facultad de Ciencias del Medio Ambiente y Centro de Investigación de la Biodiversidad y Cambio Climático, Universidad Tecnológica Indoamérica, Quito, Ecuador; 11 Instituto Patagónico para el Estudio de los Ecosistemas Continentales IPEEC- CONICET, Puerto Madryn, Argentina; 12 Laboratory of Microbiology, Wageningen University and Research, Wageningen, Netherlands; 13 Programa de Diversidade Biológica e Conservação nos Trópicos, Universidade Federal de Alagoas, Maceió, Alagoas Brazil; 14 Kunhã Asé Network of Women in Science, Salvador, Bahia Brazil; 15 Facultad de Ciencias Exactas y Naturales, Universidad Nacional de Cuyo - IANIGLA Consejo Nacional de Investigaciones Científicas y Tecnológicas (CONICET), Mendoza, Argentina; 16 Fundación Instituto Leloir, IIBBA-CONICET, Buenos Aires, Argentina; 17 Instituto Tecnológico Chascomús (INTECH, CONICET-UNSAM), Chascomús, Argentina; 18 University of Texas at Austin, Austin, TX USA; 19 División Plantas Vasculares, Museo de La Plata, Universidad Nacional de la Plata - CONICET, La Plata, Argentina; 20 Natural History Museum, London, UK; 21 Universidad Nacional de Mar del Plata, Mar del Plata, Argentina; 22 Instituto de Agrobiotecnología del Litoral (CONICET-UNL) - FBCB (UNL), Santa Fe, Argentina; 23 Sainsbury Laboratory Cambridge University, Cambridge, UK; 24 Institute of Biosciences, Biotechnology and Translational Biology (iB3), School of Natural and Exact Sciences, University of Buenos Aires - CONICET, Buenos Aires, Argentina

**Keywords:** Careers, Philosophy, Policy

## Abstract

Mentor relationships are crucial to retention, success, and wellbeing of women and underrepresented minority scientists in academia. A network of diverse mentors may support achieving long-term career goals, advancement, and retention of both mentors and mentees, thus enhancing diversity, equity, and inclusion initiatives.

## Diversified mentorship is essential to break systemic bias and make effective retention efforts

Multiple efforts have been made by institutions, societies, and organizations to offset gender disparity and exclusion of underrepresented minority scientists (URMs, i.e. racial/ethnic and gender groups constituting a lower proportion in the field than in the general population). Programs addressing gender bias and lack of diversity are slowly, but increasingly, addressing ingrained sexism and ableism in STEM fields and academia^1–3^. However, institutions and funding agencies should continue to invest in minimizing gender-specific obstacles; for example, promoting work-life balance; equalizing salaries, promotions, and start-up packages. Institutions must also address cultural taxation, discrimination, and isolation of URMs^4–6^. Further, institutions should act as tools of social justice by acknowledging women and URMs in leadership positions where they were historically ignored; honoring them with awards and nominations; and promoting and compensating them when serving as role models and mentors. Here, we recommend tangible action focused on the latter.

Mentor relationships are crucial to retention, success, and wellbeing of women and URMs in academia. Mentoring not only provides professional support, guidance, information, and advice; mentoring also offers templates of the behaviors that are needed to achieve success in the field^7,8^. Effective mentorship requires a unique personal relationship between individuals at different career stages, with the potential of benefiting the career advancement of both the mentor and the mentee^9^. Further, mentorship is distinct from supervision: “Mentoring is an opportunity to connect meaningfully with individuals in service to their pursuit of personally-defined goals or aspirations”^10^. Thus, mentorship is not automatically achieved in a supervisor-supervisee relationship^11^. However, even when dyadic mentor-mentee relationships have been shown highly beneficial^12^, the traditional model of mentoring can be insufficient to ensure retention, success, and wellbeing of underrepresented groups (by either race/ethnicity or gender identity in academia)^13–15^.

Women and URMs face many obstacles during their academic careers, in great part due to ingrained institutional disparity: decreased recognition in publications, fewer citations of papers authored by them, lower research funding allocations, and greater academic and domestic responsibilities^4,16–20^. These biases lead to gender and ethnic underrepresentation in academia, particularly at senior levels^21,22^. Indeed, women and URMs are less represented at later stages in the career path (a phenomenon known as ‘the leaky pipeline’^23^).

A network of diverse mentors may support achieving long-term career goals, advancement, and retention of both mentors and mentees, thus synergistically enhancing diversity, equity, and inclusion (DEI) initiatives and perpetuating a virtuous cycle of enriched mentoring^8^. Programs focusing on building diverse mentorship networks have been effective in promoting the success of women and other URMs in Science, Technology, Engineering and Mathematics (STEM) fields^24–27^. Cross-cultural mentoring relationships have been particularly successful when they included strategies to foster mentors’ awareness, sensitivity, and commitment to their mentees^15^. In this context, improved culturally and socially diverse mentorship can enable institutions to effectively increase DEI and overcome bias in STEM fields and academia. While multiple organizational efforts are focused on increasing DEI, the actual practice of these efforts is challenging. To achieve the goal of increased DEI, we suggest that institutions aim to: (1) emphasize mentoring strategies to increase DEI, especially those that address gender bias and URM exclusion, (2) promote multi-mentor programs and acknowledge their utility in increasing DEI, and (3) reference examples of diverse, institutional mentoring efforts.

## Multi-mentor systems have potential to enhance retention and DEI

Having diverse role models and mentors can be transformative and enhance academic performance, especially for women and URM mentees. Retention, motivation, and persistence in STEM fields and academia can be positively influenced by mentorship^25^. This is particularly impactful when female students and URMs are introduced, at an early undergraduate stage, to role models and mentors by whom they feel represented^26,28^. For instance, different mentorship programs focused on equity and inclusion of URMs have proven to be effective long after the relationship ended, sustaining the retention and success of underrepresented scientists^5,25,28–30^.

For mentorship to be impactful for academic advancement though, the individuals in the relationships cannot be separated from their cultural and societal backgrounds. Mentors and mentees pairing often reflect unconscious biases^31^ (e.g., majority-culture mentors interacting with mentees with ethnic, religious or gender affinities in ways that reflect majority-culture priorities rather than tailoring mentoring to these URM mentees’ priorities). Thus, active efforts from institutions are required to counteract the systemic inequities. Such efforts, particularly when formalized as programs, expand opportunities for both mentors and mentees to be more productive and innovative^8,31^. For example, women usually devote a substantial part of their time, more than men, to mentorship and teaching^27^. This imbalance can reduce publication and grant success which then contributes to unequal attrition of women mentors. Further, if women and URMs are less represented in advanced career stages, diverse mentors and whole lines of research are less present in STEM, which increases the burden on the available mentors even more. A pyramidal structure toward less representation of women and URMs in senior positions thereby undermines efforts to overcome mentorship and research biases while exacerbating the burden on those few women and URMs who are retained long enough to become mentors^9,13,14,19,22,23,27,29^.

**Fig. 1:**
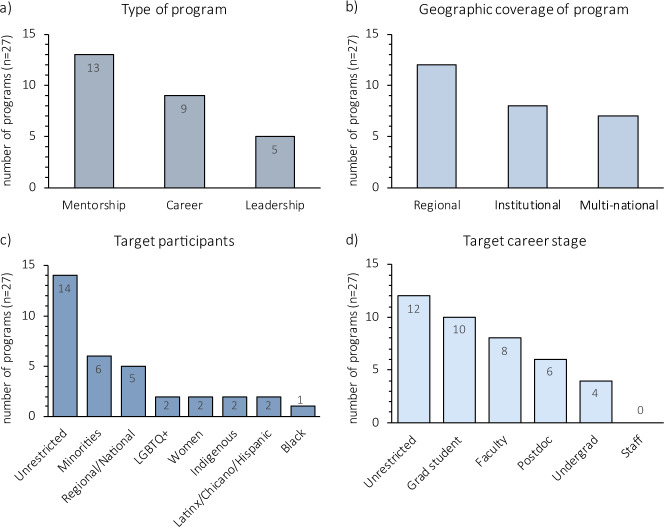
Attributes of career development, leadership and mentorship programs from around the world. Graphs represent the number of identified programs according to **a** type of program, **b** geographic coverage of the program, **c** target participants, and **d** target career stage. Most programs we reviewed focus on mentorship, are offered regionally, are not limited to specific target groups, and emphasize faculty and postdoc career stages. Notably, no programs specifically target staff (non-faculty, university employees), despite evidence that staff and contingent faculty roles (e.g., adjuncts, non-tenure-track) do extensive service in mentoring and provision of DEI services in academia. Also, most programs include multiple target categories, so totals are >n in figure sections **c** and **d**.

One solution is promoting mentorship networks, involving multiple mentor-mentee relationships, to increase collective performance by magnifying resources^8,24,32,33^. In effective mentorship networks, mentorship extends beyond the relationships of mentors and their direct mentees. Indeed, mentors can be purposely picked outside the mentee’s direct advisory network^11^. Building a multi-mentor network is an emergent property of other available systems, as programs often center around matching mentor/mentee pairs (Table 1). Such programs frequently offer the possibility of establishing peer and group mentoring relationships (for example, National Research Mentoring Network^34^), thereby building networks for people in both shared and different career stages. However, we note a gap in formal programs: resources and recognition are needed for establishing these multi-mentor networks (Table 1 and Fig. 1). First, effective mentorship programs require changing institution-wide practices and interactions to challenge systemic inequalities on all organizational levels^35^. For example, institutional policies can mitigate the overburden of mentoring by women and URMs (which, as noted, exacerbates attrition) by establishing review and promotion guidelines and responsibility re-allocation to encourage and credit mentors who participate in diverse mentoring programs^36–38^. While some solutions can be generalized, each institution faces unique circumstances that require specific actions. Although doing so is challenging, not addressing these issues in local contexts may cause even the best program to fail.

**Table 1:** Career development, leadership and mentorship programs: examples from around the world.

Program	Institution, organization, or society	Link	Purpose	Coverage	Target	Career stage
Career development	Life Sciences Career Development Society	https://lscds.org/events/mentorship-program/	Matches mentees with industry mentors, career transition to industry coaching, networking	Institutional (University of Toronto, Canada)	Open to all participants	Graduate students, postdocs, research associates (Life Sciences)
	Inclusive Graduate Education Network	https://www.igenetwork.org/	Graduate school application mentoring and coaching	Regional (North America)	Minorities – Black, Latinx and Indigenous	Graduate students (Physics)
	Chinese University of Hong Kong Mentorship Program	https://cpdc.osa.cuhk.edu.hk/student/programmes-workshops/CUMP	Matches mentees with professional and senior executive mentors, career transition to industry coaching, networking	Institutional (Chinese University of Hong Kong, Hong Kong)	Open to all participants	Undergraduate and graduate students
	Global Mentorship Program University of Nottingham	https://www.nottingham.edu.cn/en/business/career-development/global-mentorship-programme.aspx	Matches mentees with professional mentors, career transition to industry coaching, networking	Regional (University of Nottingham in China, with international mentors)	Open to all participants	Undergraduate and graduate students
	The ASPET Mentoring Network^24^	https://www.aspet.org/aspet/education-careers/aspet-programs/aspet-mentoring-network	Professional development training and coaching, group mentoring, networking	Regional (North America)	Open to all participants	Graduate students, postdocs
	Österreichische Akademie der Wissenschaften Mentoring Program	https://www.oeaw.ac.at/index.php?id=3294&L=3	Matches mentees with experienced academic mentors, career development training, networking	Regional (Austrian Academy of Sciences affiliated institutions)	Open to all participants	Graduate students, postdocs and junior faculty
	Gordon Research Conferences & Workshops	https://www.grc.org/	Professional development training	Multi-national (North America, Asia, Europe)	Open to all participants	Unrestricted
	Yale Young African Scholars – Yale University	https://africanscholars.yale.edu/	Academic skills development, college application coaching, networking	Multi-national (African countries)	Open to all African secondary students	Secondary students
	The Council for At-Risk Academics (CARA)	https://www.cara.ngo/	Matches fellows with academic mentors, funding support, advocacy	Multi-national (Syria, Zimbabwe, Iraq)	Open to academics from at-risk countries	Unrestricted
Leadership	eLife Innovation Leaders Program	https://elifesciences.org/labs/ea8e2f51/introducing-innovation-leaders-2020	Matches mentees with industry and academic mentors, group mentoring, professional and project development training, networking	Multi-national (unrestricted)	Open to all participants	Unrestricted
	LILANUT Leadership in Nutrition for LatAm Program^49^	https://www.slaninternacional.org/lilanut/sobre_nosotros.php	Professional development and leadership workshops, networking	Multi-national (Latin America)	Open to all participants	Early career researchers (Public Health Nutrition and Nutrition Related Health Policy)
	Interamerican Task Force OEA	http://www.oas.org/es/taskforcewomenleadership/	Leadership training and communication, networking, policy making	Multi-national (Latin America)	Women	Unrestricted
	SACNAS	https://www.sacnas.org/	Professional development training, leadership promotion, networking, DEI advocacy and policy making	Regional (North America)	Minorities - Chicanos/Hispanics and Native Americans	Unrestricted
	The World Bank – XL Africa	https://www.xl-africa.com/	Matches mentees with industry mentors, leadership and entrepreneurship promotion, funding support, networking	Multi-national (African countries)	African entrepreneurs	Unrestricted
Mentorship	The African Academy of Sciences	https://www.aasciences.africa/mentorship-scheme	Matches mentees with professional mentors, professional development and leadership training, networking	Multi-national (African countries)	Open to all applicants affiliated to African universities or research institutions	Postdocs and junior faculty
	Public Health Policy - Leadership/Mentorship Program for Latin American women	https://www.comunidadmujer.cl/liderazgo/programa-mentoria/	Matches mentees with industry and academic mentors, Group mentoring, Leadership training, Networking	Regional (Latin America)	Women	Unrestricted
	Center for the Improvement of Mentored Experiences in Research (CIMER)	https://cimerproject.org/	Training for individuals, institutions and organizations, Mentor and mentee training, Mentoring research, Networking, DEI advocacy	Institutional (CIMER affiliated institutions, North America)	Open to all participants affiliated to partner institutions	Unrestricted
	National Research Mentoring Network^34^	https://nrmnet.net/about-nrmn-2/	Matches mentees with mentors, Mentor and mentee training, Group mentoring, Networking, DEI advocacy	Regional (North America)	Minorities	Unrestricted
	University of Minnesota - Clinical and Translational Science Institute - Mentor Training	https://www.ctsi.umn.edu/education-and-training/mentoring/mentor-training	Mentor training	Institutional (University of Minnesota, USA)	People with faculty appointments at the university	Faculty
	Center for the Integration of Research, Teaching and Learning - Mentoring Workshops	https://www.cirtl.net/events/949	Mentor training	Institutional (Affiliated institutions)	Open to all participants affiliated to member institutions with affiliations to institutions member of the network	Graduate students, postdocs
	University of Michigan - Rackham Graduate School - Mentoring and Advising Resources	https://rackham.umich.edu/faculty-and-staff/facilitating-academic-success/mentoring-advising/ https://rackham.umich.edu/faculty-and-staff/resources-for-directors/mentoring/	Mentor and mentee training, group mentoring, networking	Institutional (University of Michigan, USA)	People with appointments at the university	Graduate students and faculty
	Hong Kong Baptist University - Mentoring Programs	https://sa.hkbu.edu.hk/career/career-programmes/mentoring-for-success https://sa.hkbu.edu.hk/career/seed/mentorship/group-mentoring	Matches mentees with professional mentors, group mentoring, networking	Institutional (Hong Kong Baptist University, Hong Kong)	People with appointments at the university	Graduate students and faculty
	The University of Hong Kong - Mentorship Programs	https://www.mentorship.hku.hk/daao/	Matches mentees with professional mentors, group mentoring, networking	Institutional (The University of Hong Kong, Hong Kong)	People affiliated or with appointments at the university	Undergraduate and graduate students, faculty
	Leopoldina Nationale Akademie der Wissenschaften - Mentoring Program	https://www.leopoldina.org/en/funding/leopoldina-fellowship-programme/mentoring-program/	Matches mentees with professional mentors, mentor and mentee training, career development training, long-term support of scholarship holders	Regional (Austria, Germany and Switzerland)	Applicants from Austria, Germany and Switzerland	Postdocs
	Mentoring Hessen - Frauen in Wissenschaften und Wirtschaft	https://www.mentoringhessen.de/internationals-en/	Matches mentees with professional mentors, career development training	Regional (German institutions)	Women	Unrestricted
	oSTEM	https://www.ostem.org/	Matches mentees with professional mentors, career development and leadership training, networking	Regional (USA)	LGBTQ + people	Unrestricted
	Out to Innovate	https://www.noglstp.org	Matches mentees with mentors, mentor and mentee training, group mentoring, networking, DEI advocacy	Regional (North America)	LGBTQ + people	Unrestricted

In this non-exhaustive list of career development, leadership, and mentorship programs from around the world, programs were identified by coauthors within their institutions, funding agencies, known publications, collaboration networks and their close scientific community. Our goal was to identify an illustrative range of programs across geographic regions, not to conduct an exhaustive inventory and analysis (though that is an area ripe for further research). Each program was classified according to the overall goal (Career development, Leadership, and Mentorship) and assessed based on the activities (Purpose), breadth (Coverage), and targets (Target and Career stage) after evaluating the available information. Purpose briefly indicates the aim of the programs. Coverage indicates the area of influence: ‘institutional’ indicates limited to institutionally affiliated staff; ‘regional’ indicates area of influence of the institution/program (up to a few countries); and ‘multi-national’ indicates reach of more than four countries of influence or not restricted to nationality. Target indicates focal participants: women, URMs, institutional staff, or unrestricted (‘open to all participants’). Career stage indicates career level for focal participants. We cite published outcomes of listed programs, if available.

Furthermore, once efforts and programs are established, their methods and outcomes must be continuously monitored and evaluated to ensure institutions reassess successful or failed activities dynamically and responsively. Effectiveness may also be achieved when pre-established programs are synchronized and interconnected, thereby helping to identify diverse mentors for specific aspects of academic life. Successful programs will offer continuous support throughout the academic path, enabling professional development of both mentors and mentees and empowering diverse groups by promoting access to available resources and providing appropriate recognition.

## Building networks enhances institutional efforts and promotes inclusivity

Building a diverse and inclusive network helps promote a sense of identity and community that counteracts feelings of isolation often experienced by scientists throughout their careers^39^. Mentoring networks, including peer mentors, should also be encouraged because they are more balanced in power and transparency, and less hierarchical^40,41^. Peer mentor networks can be particularly effective for tackling same-career-stage challenges and opportunities^28,33,42^. All these efforts should be accompanied by human resources training and implementing codes of conduct detailing best practices to make the whole experience for mentors and mentees more enjoyable and successful^36,43,44^. By diversifying the academic community with which mentors and mentees actively engage, scientific advancement can be achieved in a nurturing environment that can have a strong, positive impact on productivity^25,32,33^. Therefore, fostering mentorship networks should be a high priority for any academic institution undertaking DEI efforts.

Though not widely adopted, there are models for this approach worldwide (Table 1). Select academic institutions, professional societies, and conference organizers offer training programs to promote successful mentorship relationships that subsequently support diverse and more inclusive work environments. Although the outcomes of many programs have not yet been quantified or evaluated, some programs have minimized gender-specific barriers and reduced exclusion of URMs^24,45–47^. Evaluating outcomes of mentorship programs remains challenging; traditional quantitative approaches may not be sufficient to capture how programs promote careers in nuanced, individualized ways that transcend the normative metrics of academic productivity and research impact (which themselves can promote inequalities)^48^. Even though assessment for such programs is challenging, the outcomes can be qualified within a range of acceptable parameters assessing individual, group, and institutional growth^24,45–47^. Since women and URM scientists are generally more involved in formal and informal mentoring activities^27^, overburdening may be detrimental for program implementation and success. Therefore, evaluations should be accompanied by incentive programs for mentors, such as release from administrative and departmental duties and provision of funding awards. Funding agencies should also: (1) explicitly include DEI requirements in their guidelines for grant spending, (2) provide resources to support reviewers, grantees, and institutions in the implementation of mentoring networks that could arise within institutions or from collaboration networks, and (3) hold grantees accountable if not following those guidelines.

## Steps forward

Policy changes and active implementation of programs that promote effectively, diverse mentorship networks can help women and URMs to fully achieve their potential as scientists. And yet, we see a disheartening lack of resources directed to career development, leadership, and mentorship programs in some regions (Fig. 1). For example, our own experiences and observations indicate there is a particular dearth of these resources across the Global South (i.e. Latin America, Africa, and South Asia; Fig. 1). Indeed, other than collaboration groups or a few institutional efforts, many academics worldwide have no support to successfully navigate the mentoring process, establish healthy relationships with their mentors or mentees, or even have a resource to which they can turn should problematic situations arise.

**Fig. 2:**
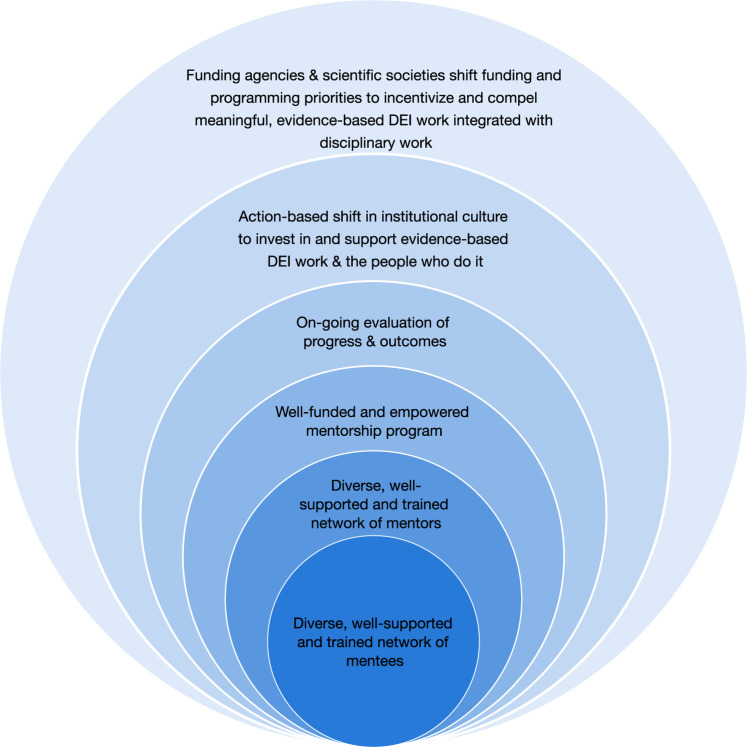
Framework to build diverse multi-mentors networks. Establishing effective programs that promote diverse multi-mentor networks -- in which mentees and mentors are supported and trained to (1) build healthy relationships, (2) develop professionally, and (3) achieve success – is a layered process that depends on many embedded factors: **a** DEI assessment implemented by institutions, funding agencies, scientific societies, and the community; **b** policy and work culture changes to promote evidence-based DEI work; **c** continuous support and evaluation of program implementation, progress, and outcomes; and **d** reassessment, adaptation, and reevaluation of programs to empower mentorship networks. As a dynamic system, all components feed from and are informed by the others.

A diverse environment improves working and learning experiences for the people involved, brings new perspectives to research, encourages more people to work in STEM fields and academia, and improves opportunities for everyone involved. To achieve this goal, we urge all agencies, foundations, institutions, and societies to use the many existing examples to inform their development of programs to generate effective mentoring relationships within their sphere of influence. We also present a step-by-step framework to diversify and build mentorship networks that includes all the aspects discussed in this commentary (Fig. 2). Building effective, diverse, multi-mentor networks can only work when programs are informed and accompanied by institution-wide DEI policy implementation, reflect diversity of the community in which institutions are embedded, and synchronize support for all organizational and career levels. Healthier work environments, more inclusive science training, and better outcomes for professionals from all backgrounds would ideally ensue as successful program implementation broadens, creating a virtuous cycle of enriched mentoring for future generations.

## References

[1] Moss-Racusin CA (2016). A “Scientific Diversity” intervention to reduce gender bias in a sample of life scientists. CBE Life Sci. Educ..

[2] Kong S, Carroll K, Lundberg D, Omura P, Lepe B (2020). Reducing gender bias in STEM. MIT Sci. Policy Rev..

[3] Best KL, Sanwald U, Ihsen S, Ittel A (2013). Gender and STEM in Germany: policies enhancing women’s participation in academia. Int. J. Gender Sci. Technol..

[4] Salinas C, Riley P, Camacho L, Floyd DL (2020). Mentoring experiences and perceptions of latino male faculty in higher education. Hispanic J. Behav. Sci..

[5] Freeman JB (2020). Measuring and resolving LGBTQ disparities in STEM. Policy Insights Behav. Brain Sci..

[6] Greider CW (2019). Increasing gender diversity in the STEM research workforce. Science.

[7] Lockwood P (2006). "Someone like me can be successful": do college students need same-gender role models. Psychol. Women Q..

[8] Montgomery, B. L. & Page S. C. *Mentoring beyond Hierarchies: Multi-Mentor Systems and Models*. Commissioned Paper for National Academies of Sciences, Engineering, and Medicine Committee on Effective Mentoring in STEMM, 1–25 (2018).

[9] Kalbfleisch, P. & Keyton, J. *Gender, Power, and Communication in Human Relationships* (Routledge, 2012).

[10] Montgomery, B. L. *Living My Purpose In Multiple Domains And Cherishing Every Moment*. http://www.berondamontgomery.com/.

[11] Mellon A, Murdoch-Eaton D (2015). Supervisor or mentor: is there a difference? Implications for paediatric practice. Arch. Dis. Child..

[12] Malmgren RD, Ottino JM, Nunes Amaral LA (2010). The role of mentorship in protégé performance. Nature.

[13] Ibarra, H., Carter, N. M. & Silva, C. Why Men Still Get More Promotions Than Women. *Harvard Business Review* 8 (2010). 20821967

[14] Dunham, C. C., Weathers, L. H., Hoo, K. & Heintz, C. I just need someone who knows the ropes: mentoring and female faculty in Science and Engineering. *J. Women Minorities Sci. Eng.* **18**, 79–96 (2012).

[15] Kent M, A., Kochan F, M. Green A (2013). Cultural influences on mentoring programs and relationships: a critical review of research. Int. J. Mentor. Coaching Educ..

[16] Budrikis Z (2020). Growing citation gender gap. Nat. Rev. Phys..

[17] Astegiano J, Sebastián-González E, Castanho C (2019). de T. Unravelling the gender productivity gap in science: a meta-analytical review. R. Soc. Open Sci..

[18] Oliveira DFM, Ma Y, Woodruff TK, Uzzi B (2019). Comparison of National Institutes of Health grant amounts to first-time male and female principal investigators. JAMA.

[19] Diele-Viegas, L. M. et al. Potential solutions for discrimination in STEM. *Nat. Hum. Behav.*10.1038/s41562-021-01104-w (2021). 10.1038/s41562-021-01104-w 33875839

[20] Broderick NA, Casadevall A (2019). Gender inequalities among authors who contributed equally. eLife.

[21] Monroe K, Ozyurt S, Wrigley T, Alexander A (2008). Gender equality in academia: bad news from the trenches, and some possible solutions. Perspect. Politics.

[22] Ceci SJ, Williams WM (2011). Understanding current causes of women’s underrepresentation in science. Proc. Natl Acad. Sci. USA.

[23] Gasser CE, Shaffer KS (2014). Career development of women in academia: traversing the leaky pipeline. TPC.

[24] Womack VY (2020). The ASPET mentoring network: enhancing diversity and inclusion through career coaching groups within a scientific society. LSE.

[25] Estrada M, Hernandez PR, Schultz PW (2018). A longitudinal study of how quality mentorship and research experience integrate underrepresented minorities into STEM careers. LSE.

[26] Hernandez PR (2020). Inspiration, inoculation, and introductions are all critical to successful mentorship for undergraduate women pursuing geoscience careers. Commun. Earth Environ..

[27] Misra, J., Lundquist, J. H., Holmes, E. & Agiomavritis, S. The ivory ceiling of service work. *Am. Assoc. Univ. Profr.* https://www.aaup.org/article/ivory-ceiling-service-work (2011).

[28] Hernandez PR (2017). Promoting professional identity, motivation, and persistence: Benefits of an informal mentoring program for female undergraduate students. PLoS ONE.

[29] Hughes BE (2018). Coming out in STEM: factors affecting retention of sexual minority STEM students. Sci. Adv..

[30] Mentorship Structures: What Forms Does Mentorship Take? *Natl Acad. Sci. Eng. Med.*10.17226/25568 (2019).

[31] Malone, S. L. & Record, S. Addressing bias in faculty retention. *Ecol. Appl.* **31**, e02346 (2021). 10.1002/eap.2346 34181313

[32] Nielsen MW (2017). Opinion: gender diversity leads to better science. Proc. Natl Acad. Sci. USA.

[33] Woolley AW, Aggarwal I, Malone TW (2015). Collective intelligence and group performance. Curr. Dir. Psychol. Sci..

[34] Sorkness CA (2017). A new approach to mentoring for research careers: the National Research Mentoring Network. BMC Proc..

[35] Dennissen M, Benschop Y, van den Brink M (2019). Diversity networks: networking for equality?. Br. J. Manag..

[36] Lunsford LG, Baker V, Griffin KA, Johnson WB (2013). Mentoring: a typology of costs for higher education faculty. Mentor. Tutor..

[37] Schwartz J (2012). Faculty as undergraduate research mentors for students of color: taking into account the costs. Sci. Educ..

[38] Morrison, J. A. et al. *Recognizing and Valuing the Mentoring of Undergraduate**Research, Scholarship, and Creative Activity by Faculty Members: Workload, Tenure, Promotion, and Award Systems. CUR White Paper No. 2*. *Online Submission* (2019).

[39] Cameron, K. A., Daniels, L. A., Traw, E. & McGee, R. Mentoring in crisis does not need to put mentorship in crisis: realigning expectations. *J. Clin. Transl. Sci.* **5**, 1–8 (2021).

[40] Tran NA (2014). The role of mentoring in the success of women leaders of color in higher education. Mentor. Tutor..

[41] Akinla O, Hagan P, Atiomo W (2018). A systematic review of the literature describing the outcomes of near-peer mentoring programs for first year medical students. BMC Med. Educ..

[42] Dennehy TC, Dasgupta N (2017). Female peer mentors early in college increase women’s positive academic experiences and retention in engineering. Proc. Natl Acad. Sci. USA.

[43] Hund AK (2018). Transforming mentorship in STEM by training scientists to be better leaders. Ecol. Evol..

[44] Whittaker JA, Montgomery BL, Martinez Acosta VG (2015). Retention of underrepresented minority faculty: strategic initiatives for institutional value proposition based on perspectives from a range of academic institutions. J. Undergrad. Neurosci. Educ..

[45] Rogers J, Branchaw J, Weber-Main AM, Spencer K, Pfund C (2020). How much is enough? The impact of training dosage and previous mentoring experience on the effectiveness of a research mentor training intervention. Underst. Interv..

[46] House SC, Spencer KC, Pfund C (2018). Understanding how diversity training impacts faculty mentors’ awareness and behavior. IJMCE.

[47] Guerrero LR (2017). Using collaborative approaches with a multi-method, multi-site, multi-target intervention: evaluating the National Research Mentoring Network. BMC Proc..

[48] Rhaiem M (2017). Measurement and determinants of academic research efficiency: a systematic review of the evidence. Scientometrics.

[49] Latin VII (2018). American Workshop on Leadership in Nutrition. Proposal and Actions to Decrease Malnutrition in Latin America and the Caribbean. Food Nutr. Bull..

